# The incomparability of cause of death statistics under “one country, two systems”: Shanghai versus Hong Kong

**DOI:** 10.1186/s12963-017-0155-z

**Published:** 2017-09-29

**Authors:** Jiaying Zhao, Edward Jow-Ching Tu, Chi-kin Law

**Affiliations:** 1 The Institute for Asian Demographic Research, School of Sociology and Political Science, Shanghai University, Shanghai, China; 2 School of Demography, The Australian National University, Canberra, Australia; 3 Asia Population Forum and Retired Faculty, School of Humanities and Social Science, Hong Kong University of Science and Technology, Clear Water Bay, Hong Kong; 4 Centre for Applied Health Economics, School of Medicine, Menzies Health Institute Queensland, Griffith University, Nathan, Australia; 5 The Jockey Club School of Public Health and Primary Care, The Chinese University of Hong Kong, Sha Tin, Hong Kong

## Abstract

**Background:**

Valid and comparable cause of death (COD) statistics are crucial for health policy analyses. Variations in COD assignment across geographical areas are well-documented while socio-institutional factors may affect the process of COD and underlying cause of death (UCD) determination. This study examines the comparability of UCD statistics in Hong Kong and Shanghai, having two political systems within one country, and assesses how socio-institutional factors influence UCD comparability.

**Methods:**

A mixed method was used. Quantitative analyses involved anonymized official mortality records. Mortality rates were analyzed by location of death. To analyze the odds ratio of being assigned to a particular UCD, logistic regressions were performed. Qualitative analyses involved literature reviews and semi-structural interviews with key stakeholders in death registration practices. Thematic analysis was used.

**Results:**

Age-standardized death rates from certain immediate conditions (e.g., septicemia, pneumonia, and renal failure) were higher in Hong Kong. Variations in UCD determination may be attributed to preference of location of death, procedures of registering deaths outside hospital, perceptions on the causal chain of COD, implications of the selected UCD for doctors’ professional performance, and governance and processes of data quality review.

**Conclusions:**

Variations in socio-institutional factors were related to the process of certifying and registering COD in Hong Kong and Shanghai. To improve regional data comparability, health authorities should develop standard procedures for registering deaths outside hospital, provide guidelines and regular training for doctors, develop a unified automated coding system, consolidate a standard procedure for data review and validity checks, and disseminate information concerning both UCD and multiple causes of death.

## Background

Valid and comparable cause of death (COD) statistics are essential to identify emerging public health challenges, to inform and assist in priority setting and health services planning, and to evaluate health policies and interventions [1, 2]. To ensure the uniformity and quality of mortality data for valid comparisons across regions, the World Health Organization (WHO) has advocated a standardized COD diagnosis form for death certification [3].

CODs are reported in death certificates, which consist of two parts. Part I includes a chain of events leading directly to death, with the immediate COD (the final disease, injury, or complication directly causing death) stated on the first line and the underlying cause of death (UCD) on the last [4]. UCD is defined as “the disease or injury which initiated the train of morbid events leading directly to death” [5] (vol 2: p 31). Part II includes all other significant diseases, conditions, or injuries that contributed to the death but did not result in the UCD given in Part I [4]. COD data are usually compiled and disseminated in the form of yearly statistics on the basis of UCD under the Tenth Revision of the International Classification of Diseases and Related Health Problems (ICD-10), a medical classification list by WHO [5].

Subject to differences in physicians’ death certification practices, previous studies showed that the rules of UCD determination for several CODs are not consistent across different death registration systems, making comparisons difficult [6, 7]. For example, in a work by Reid and Rose (1964) [8], British doctors were found to be more likely to certify the term “bronchitis” than American doctors when certifying the same deceased case. Such differences might explain why reported bronchitis mortality in England and Wales was 44 times higher than that in America during the late 1950s. Similar analyses were conducted in examining reporting practices for cancer [9, 10], chronic obstructive pulmonary disease [10], and diabetes [7, 11, 12]. The lack of comparability in COD assignment may give rise to misinterpretation regarding the epidemiologic changes at local, regional, and global levels [2, 13], leading to the distortion of health care system planning and policy development.

UCD statistics originate from death certificates, an administrative product affected by the formal and legal aspects of government structures (i.e., institutional settings). These institutional factors may influence the professional performance of doctors and procedures for certifying and coding UCD. Consequently, procedures adopted by different health care systems may lead to inconsistency in UCD determination in different areas. Also, social contexts may have an impact on death registration practices [14]. Although the incomparability of UCD across regions is well documented [7, 15], there has been scant research exploring how social and institutional factors affect UCD determination through the process of data initiations, collections, coding, and audits [16]. This paper aims to fill that gap.

Specifically, this study aims to examine the comparability of UCD statistics in Hong Kong and Shanghai, to assess the effect of social and institutional factors on the comparability of UCD statistics, and to suggest possible solutions to improve the data comparability across countries and regions. These two cities are among the most developed areas in China, which has experienced one of the most remarkable mortality declines in the last 30 years. In 2010, life expectancy of both cities was above 82 years [17], with similar age-specific mortality rates for the period of 2005–2007 (see Table 1). According to the 2013 update of the Global Burden of Disease (GBD) study, both cities were categorized as having low levels of mortality with the standards of high-income economies [18]. In 2008, the numbers of physicians per 10,000 persons in Hong Kong and Shanghai were 18 and 27, respectively. Despite the above similarities, both cities have been under different political and ruling systems since the mid-19^th^ century, which may influence the physicians’ and coders’ perceptions and practices in certifying death certificates, as well as data collection and coding practices of the death registration systems. All of these may lead to the incomparability of UCD statistics between the two cities, as suggested by Zhou and colleagues in the GBD study of 2013 [18]. For example, age-standardized rates of death (per 100,000 people) for lower respiratory infections in Hong Kong (82.3 for males, 59.2 for females) were consistently higher than in Shanghai (6.3 for males, 4.0 for females), and even in Tibet (53.1 for males, 42.8 for females).

**Table 1:** Comparison of institutional structures of death registration systems in Hong Kong and Shanghai

Data compilation process		Hong Kong	Shanghai
Coverage	Compulsory registration of death events	Yes	Yes
	Required time for registration after death	24 hours	Before cremation
	Coverage	De-facto (All deaths within the territory)	De-jure (permanent residents of Shanghai based on household register)
	Number of physicians per 10,000 population in 2008	18	27
Data initiation	Who certifies death for those dying in hospital	Attending doctors	Attending doctors
	Who certifies death for those dying outside hospital without a doctor attending	Coroners	Non-criminal: community doctors; Criminal: police
COD^a^ coding	Coding rule of COD	ICD-10 (ACME)^b^	ICD-10 (Manual)
Data audit	Clinical record review for COD	If person dies from infectious disease; deaths with insufficient information to determine UCD	Case of death when the causal sequence is not specific or only the mechanism of death is reported
	Training for physicians to certify death	No	Yes

^a^Cause of death

^b^Automated Classification of Medical Entries

Sources of data: Shanghai Municipal Statistical Bureau, National Bureau of Statistics Survey (Shanghai Groups) (2009) [18]; The Hong Kong Council of Social Service (2016) [19]; Zhao (2013) [20].

This study will elicit the factors for UCD incomparability between death registration systems in Hong Kong and Shanghai. The institutional structures of the death registration system in the two cities are first contrasted and compared. Next, we assess the comparability of UCD through quantitative analysis. Then, opinions extracted from the informant interviews and document reviews are used to explore socio-institutional effects. Finally, possible solutions for improving data incomparability are proposed and discussed.

## Methods

### Study settings: institutional structure of the death registration system in Hong Kong and Shanghai

Although Hong Kong and Shanghai are both key financial hubs in China, the two cities have been governed by different political systems for decades, resulting in corresponding death registration systems that bear, to some degree, the legacy of past political systems. The two cities have different institutional structures, legislation, and regulations relating to the practice of death registration (Table 1) [19–21].

#### Hong Kong

As a former UK-dependent territory, the death registration system of Hong Kong is inherited from the British system. According to current legislation, every death in Hong Kong, regardless of the decedent’s residence status, has to be reported to the Births and Deaths Registration of the Immigration Department within 24 hours. A penalty is levied if registration is not filed in time.

**Fig. 1:**
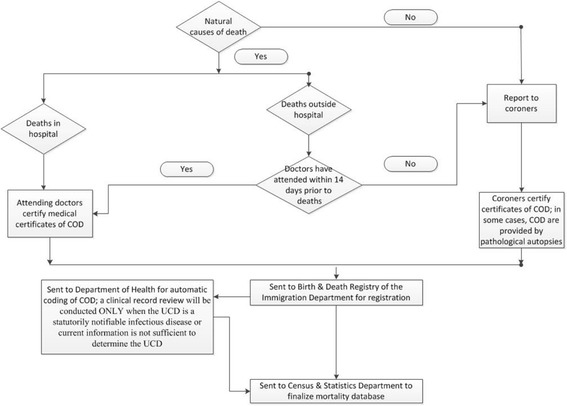
Certification and registration of cause of death in Hong Kong

The procedures for certifying and registering death vary significantly across different locations of death (see Fig. 1). If a death occurs in hospital and does not involve any external causes of death (like homicide, suicide, or an accident), the medical practitioner who attended the deceased person during his or her last illness will issue a Medical Certificate for the Cause of Death (Form 18) [22]. In completing Form 18, the medical practitioner should certify each disease, abnormality, injury, or poisoning that he/she believes has adversely affected the decedent in the death certificate, and provide information with sufficient details based on ICD-10 [23].

If a death occurs outside hospital, the case is usually reportable to the Coroner, unless an authorized medical practitioner has attended the deceased person within 14 days prior to death and has signed and issued Form 18 within 24 hours of the death [22, 24]. The procedure for certifying and registering a reportable death is more complicated. For a reportable death, the body is sent to either a hospital or a public mortuary. A forensic pathologist will conduct an external examination of the body and disclose the findings of this examination and the COD, if ascertained, to the coronial office. Based on this information, the Coroner will decide whether the reportable death should be further investigated by ordering an autopsy, a police investigation, or even a death inquest to determine the COD.

For registering a reportable death, the Coroner will directly inform the Registrar of Deaths when the COD is determined [22]. For a non-reportable death, the informant, who is always a close relative or guardian of the deceased person, is required to take the signed Form 18 to the Births and Deaths General Register Office of the Immigration Department [22]. Next, digitalized data on death records are sent to the Department of Health to conduct COD coding through Automatic Classification of Medical Entry (ACME) software. Clinical records of non-reportable deaths are not reviewed unless the UCD is a statutorily notifiable infectious disease or current information is not sufficient to determine the UCD [25].

To ensure the quality of COD statistics, guidelines are available for completing death certificates in Hong Kong [23]. However, there are no regular training programs for physicians who need to certify non-reportable deaths.

#### Shanghai

The death registration system of Shanghai was established in 1951 after the establishment of the People’s Republic of China (PRC). In contrast to the Hong Kong system, the death registration system of Shanghai is based on its household registration system (*Hukou*). The legal basis of death registration in Shanghai is enshrined in administrative regulations: any death should be reported to the local police station for deregistering the deceased person’s permanent residence within 15 days before the body is allowed to be cremated. In some instances, persons with Shanghai *Hukou* who die outside Shanghai may not be recorded. Such deaths account for about 1% of the overall deaths in Shanghai annually [21]. This may lead to slight underestimations of the genuine mortality level, as bodies may be cremated elsewhere without being registered in the Shanghai death registration system.

**Fig. 2:**
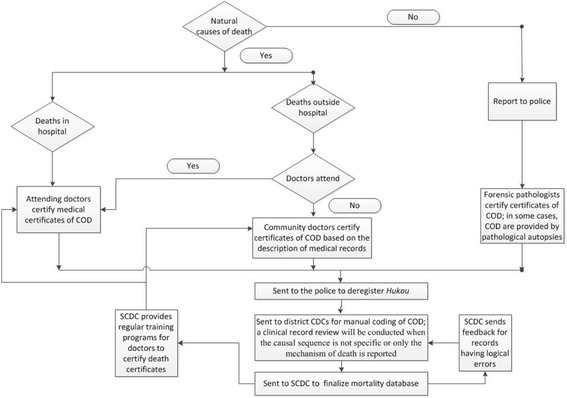
Certification and registration of cause of death in Shanghai

The procedures for certifying and registering deaths in Shanghai vary across different death locations (see Fig. 2). If a person dies in hospital without the involvement of external causes (i.e., a natural death), an attending physician will issue a death certificate. If a natural death occurs outside hospital, a community physician will issue a death certificate based on the description of clinical records, even if the community physician has never attended the deceased person. Community physicians are not requested to examine a body to determine the COD. Only when the UCD is not ascertained or is suspected to be a suicide or homicide, the case will be referred to the police for further investigation.

For registering a natural death, the informant is required to take the death certificate to the police station to deregister *Hukou.* The police then send a copy of the death certificate to the district’s Center for Disease Control and Prevention (CDC) to conduct manual coding of the COD. A clinical record review will be conducted when the causal sequence is not specific (e.g., senility) or only the mechanism of death (e.g., renal failure) is reported [21].

The quality control of COD statistics is rigorous in Shanghai [21]. Apart from the physicians’ guidelines on death certification implemented by the Shanghai Center for Disease Control and Prevention (SCDC), the SCDC provides regular training programs for physicians certifying death certificates. The quality of certifying COD is reviewed and used to evaluate physicians’ performance based on the rules set in the guidelines, owing to the bureaucratic system (i.e., SCDC, district CDC, hospitals, or community service centers).

### Methods

#### Quantitative analysis

To assess the comparability of UCD statistics across the two cities, we obtained anonymized mortality data from Hong Kong’s Department of Health (Hong Kong: for the period 2005–2008) and the SCDC (Shanghai: for the period 2005–2007). UCD is selected according to ICD-10 selection rules through manual coding in Shanghai and the Automated Classification of Medical Entries (ACME) system in Hong Kong.

Using the aggregate data, COD for each city was compared based on UCD. As noted, deaths occurring in different locations are treated differently when death certificates are issued. UCD-specific mortality rates in the two cities were analyzed by location of death. Mortality rates were age-standardized using the World Standard Population [26].

Individual death records in the two cities were used to analyze the effects of individual- (i.e., age, sex) and city-level factors on COD assignment between the two cities. Logistic regression was performed to estimate the odds ratio of a death being assigned to a particular UCD versus being assigned to another between the two cities (i.e., city-level factors), controlling the effects of age and sex (i.e., individual-level factors) [2].

#### Qualitative analysis

In addition to quantitative analyses, qualitative analyses were applied to review documents related to the death registration systems; interviews were conducted to elicit experts’ perspectives on certifying and classifying UCD in the two cities. We conducted 15 semi-structural interviews with physicians, coroners, experts on COD research, and officers in charge of death registration systems. Research participants were collected through snowball sampling. Interview topics included case review of COD, clinicians’ concerns in the completion of death certificates, procedures for collecting and coding death certificates, and processes of data quality control. Informed consent was obtained through telephoning or emailing contacts. Each in-depth interview lasted approximately 90 minutes. Thematic analysis, as noted in Braun and Clarke, was used in the present analysis [27].

## Results

### Differences in UCD distribution between Hong Kong and Shanghai

Over the period of the study, overall age-standardized death rate (ASDR) was 3.6 (per 1,000 persons) for Hong Kong and 4.1 for Shanghai (Table 2). In both cities, malignant neoplasms were the leading cause of death, accounting for more than 30% of overall ASDR.

**Table 2:** Age-standardized death rates (ASDR) and odds ratios by selected underlying cause of death (UCD) in Hong Kong (2005–2008) and Shanghai (2005–2007)^a^

UCD	ASDR				Adjusted odds ratio^c^	
	Hong Kong		Shanghai		Hong Kong vs. Shanghai	
	ASDR (per 100,000)	% of overall ASDR^b^	ASDR (per 100,000)	% of overall ASDR^b^	Estimate	95%CI
Septicemia (A40-A41)	6.3	1.8	0.02	0.0	**1,435.4**	**538.3–3,827.2**
Malignant neoplasms (C00-C97)	118.6	33.2	123.3	30.2	**1.1**	**1.1–1.1**
Diabetes mellitus (E10-E14)	4.7	1.3	15.1	3.7	**0.4**	**0.3–0.4**
Ischemic heart diseases (I20-I25)	36.0	10.1	40.2	9.9	**1.0**	**1.0–1.0**
Cerebrovascular diseases (I60-I69)	29.8	8.3	74.1	18.2	**0.4**	**0.4–0.4**
Pneumonia (J12-J18)	37.6	10.5	2.1	0.5	**28.0**	**26.5–29.5**
Chronic lower respiratory diseases (J40-J47)	17.5	4.9	38.8	9.5	**0.5**	**0.4–0.5**
Renal failure (N17-N19)	11.2	3.1	1.2	0.3	**11.2**	**10.5–12.0**
External causes (V01-Y89)	21.6	6.0	28.6	7.0	**0.6**	**0.6–0.7**
All causes	357.7	100	408.1	100	**--**	**--**

^a^Figures in bold indicate a significance level of 0.05.

^b^% of overall ASDR for a certain UCD=ASDR for the UCD/ASDR for all causes^a^100%

^c^ Adjusted by sex and age using logistic regression. Shanghai is the reference group

Data Sources:

Department of Health, HKSAR Government (Hong Kong); Shanghai Center for Disease Control and Prevention (Shanghai)

Table 2 presents statistics on UCD and ratios of the odds of being assigned to a particular UCD (adjusted by age and sex), which suggested that certain CODs are more distinctive and thus less likely to be comparable between Hong Kong and Shanghai. Compared with deaths in Shanghai (reference group), septicemia, pneumonia, and renal failure were more likely to be coded as UCDs in Hong Kong. In contrast, diabetes, cerebrovascular diseases, and chronic lower respiratory diseases were less likely to be coded as UCDs in Hong Kong compared to Shanghai. However, the regional variations between the two cities were small for malignant neoplasms (Adjusted OR=1.1, 95% CI: 1.1˗1.1) and ischemic heart disease (Adjusted OR=1.0, 95% CI: 1.0 ˗ 1.0).

Further analyses by location of death revealed that the proportion of people who died outside hospital was much lower in Hong Kong (7%) than in Shanghai (47%) over the study period. Furthermore, the distributions of UCD between the two cities varied substantially between decedents in hospital and those at other locations (i.e., outside hospital) (Table 3). In Shanghai, the proportion of deaths from cerebrovascular disease (21.7%) and chronic lower respiratory disease (11.8%) were comparatively greater among deaths outside hospital than those in hospital (cerebrovascular disease at 18.5%; chronic lower respiratory disease at 9.6%). By contrast, the proportion of deaths from pneumonia (0.4%) and renal failure (0.2%) were lower among deaths outside hospital than those in hospital (pneumonia at 0.6%; renal failure at 0.4%). But a reverse pattern for cerebrovascular disease and chronic lower respiratory disease was observed in Hong Kong.

**Table 3:** Number and proportion of deaths by selected underlying cause of death (UCD) by locations of death in Hong Kong and Shanghai

UCD	Hong Kong				Shanghai			
	At hospital		At other locations^a^		At hospital		At other locations^a^	
	Number	%	Number	%	Number	%	Number	%
Septicemia (A40-A41)	2,904	2.0	7	0.1	2	0.0	2	0.0
Malignant neoplasms (C00-C97)	46,949	32.1	2,226	20.0	58,274	36.5	32,074	22.5
Diabetes mellitus (E10-E14)	2,104	1.4	63	0.6	6,785	4.3	5,226	3.7
Ischemic heart diseases (I20-I25)	15,536	10.6	1,214	10.9	19,736	12.4	13,239	9.3
Cerebrovascular diseases (I60-I69)	13,555	9.3	385	3.5	29,450	18.5	30,826	21.7
Pneumonia (J12-J18)	18,653	12.7	303	2.7	987	0.6	526	0.4
Chronic lower respiratory diseases (J40-J47)	7,857	5.4	527	4.7	15,286	9.6	16,794	11.8
Renal failure (N17-N19)	5,100	3.5	58	0.5	598	0.4	346	0.2
External causes (V01-Y89)	4,037	2.8	3,694	33.2	6,152	3.9	11,470	8.1
All causes	146,466	100.0	11,125	100.0	159,530	100.0	142,360	100.0

^a^Include all deaths outside hospital

Data sources:

Department of Health, HKSAR Government (Hong Kong); Shanghai Center for Disease Control and Prevention (Shanghai)

### Factors influencing UCD determinations between the two cities

To elucidate the social and institutional factors causing differences in registering deaths between the two cities, qualitative data revealed four major themes. These included

- Differences in location of death;
- Physicians’ and coders’ perceptions and judgment on the causal sequence of morbid events leading to death;
- Implications of the selected UCD for the professional performance of doctors and the potential risk for medical treatment disputes; and
- Institutional factors influencing the procedure of quality control of COD statistics.

Each of the four themes was supported by illustrative quotes and presented in Table 4.

- Location of death

**Table 4:** Factors affecting UCD determinations in Hong Kong and Shanghai: Extracts from interviews

Themes	Study areas	Quotations
Location of death	Hong Kong	*“In Hong Kong, most deaths outside hospital are reportable deaths whose UCD are based on a coroner’s investigation. The procedure of certifying a death certificate for a reportable death is more complicated compared with a natural death in hospital. Also, the property value may be depreciated if a death happens in a residential property. Thus, proportion of death outside hospital in Hong Kong is lower than places where doctors can certify death certificates based on clinical history.”* (Pathologist, Hong Kong)
	Shanghai	*“It is quite common that elderly people are bedridden due to cerebrovascular disease or injury, and then die at home.”* (A community doctor, Shanghai)
Physicians’ and coders’ perceptions of causal sequence of morbid events leading to death	Hong Kong	*“I think pneumonia and sepsis are acceptable causes of death, especially as Hong Kong is an aging society, it is difficult to identify the underlying cause of death among several causes of death across a patient’s life course.”* (Medical practitioner, Hong Kong) *“Although a lot of old patients have a history of ischemic heart disease or stroke, they may die from pneumonia or septicemia during the latest admission. I am not so sure that the causal relationship between pneumonia or septicemia and an existing chronic disease. I think, in those cases, pneumonia or septicemia is the most precise cause of death…”* (Cardiologist, Hong Kong) *“… chronic heart failure, respiratory failure, pneumonia, urinary tract infections, sepsis, and chronic renal failure are the most common dying symptoms or conditions. These diseases are the most direct causes of death…. As for existing chronic diseases, such as stroke, now about one-third of cases can recover.”* (Neurologist, Hong Kong) *“Some clinic practitioners have different perceptions of cause of death from ours. They care more about how a patient dies, especially during the last stage. We work with the Department of Health to train doctors how to certify death certificates. But we still have a long way to go.”* (Clinical pathologist, Hong Kong) *“When a patient’s blood culture test result is positive, the diagnosis of sepsis is clear. I do not think that there is any problem to certify sepsis as cause of death.”* (Medical practitioner, Hong Kong)
	Shanghai	*“It is quite common for patients to die from pulmonary infection and other infections directly. But I will certify their existing chronic diseases on death certificates as well.”* (Medical practitioner, Shanghai) *“According to Rule 3, pneumonia can be a complication of any disease. We believe that to date, people usually cannot die from pneumonia without underlying chronic diseases except for the elderly and young children. Thus, these underlying chronic diseases should be the underlying cause of death.”* (Official in Shanghai CDC, Shanghai) *“We do not think that pneumonia and septicemia are specific enough as UCD.”* (Official, Shanghai Center for Disease Control and Prevention, Shanghai)
Implications of the selected UCD for the professional performance of doctors	Hong Kong	*“We are asked to identify the accurate cause of death. The causal relationship between the acute infection and existing chronic conditions is ambiguous. If the diagnosis is not accurate, this diagnosis may cause some trouble.”* (Medical practitioner, Hong Kong)
	Shanghai	*“The relationships between doctors and patients are intensive in these years. If a patient dies from serious chronic diseases, it can be accepted by their family. But if a patient dies from infections in hospital, we may be blamed by some patients’ families. … Nosocomial infection is quite common. Why do we need to put ourselves in trouble?”* (Cardiologist, Shanghai)
Institutional influence on the procedure of quality control of cause of death statistics	Hong Kong	*“A lot of death certificates were only certified pneumonia or sepsis, and some cases mentioned chronic diseases in Part II. It is very common in Hong Kong. We code UCD based on the information from death certificates. … We usually do not review clinical records for confidential reasons.”* (Official, Department of Health, Hong Kong)
	Shanghai	*“We do not think that pneumonia and septicemia are specific enough as UCD. So if a case only mentions pneumonia or septicemia, we will review the clinical history. From my experience, most of these cases have a more specific chronic disease which initiates the chain leading to death.”* (Medical practitioner, Shanghai)

As illustrated in Table 4, Figs. 1, and 2, practices of certifying death certificates and determining UCD vary by location of death. Differences in certifying and registering procedures for deaths outside hospital between Hong Kong and Shanghai affected people’s decisions on location of death. In Hong Kong, close relatives of a deceased person (and dying persons themselves) prefer to die in hospital, in order to avoid the complicated procedures for registering deaths occurring outside hospital (most likely to be reportable deaths) and the possibility of lowering property values. Such details were associated with the low proportion of deaths outside hospital in Hong Kong.

In Shanghai, clinical records, including information on pre-existing chronic diseases, are an important source in the determination of UCD for certifying deaths outside hospital. This may explain why chronic diseases (e.g., cerebrovascular disease, chronic lower respiratory disease) were more likely to be coded as UCD in Shanghai, particularly for deaths outside hospital, than in Hong Kong.

(b). Physicians’ and coders’ perceptions of causal sequence of morbid events leading to death

Physicians and coders in the two cities had different perceptions of the causal relationship of morbidity events. They held different opinions on whether an immediately terminal condition should be determined as UCD. This may partly explain the variations in COD between the two cities. In Hong Kong, both general practitioners and specialists tended to hold the view that an immediate condition (e.g., pneumonia, septicemia, renal failure) was an accurate cause of death and the relationship between an immediate condition and existing chronic disease was uncertain. Their views were confirmed by pathologists and officers in charge of the death registration system in Hong Kong.

In Shanghai, physicians and coders generally agreed that an immediate condition caused by an existing chronic disease was not specific enough to be defined as an UCD and that immediate conditions were mostly caused by chronic diseases. This may partly explain why a lower share of septicemia, pneumonia, and renal failure was coded as UCD in Shanghai.

(c). Social and instructional impact on the professional performance of doctors related to the selected UCD

The implications of the certified UCD may affect doctors’ views on the sequence of morbid events leading to death, and consequently influence their practices of filling out death certificates. In Hong Kong, the medical doctors were concerned about the implications for their profession when filling out death certificates. According to the existing practice, it may constitute professional misconduct if CODs stated in a death certificate were controversial and conflicting. In some instances, the Hong Kong Medical Council or related regulatory bodies may take disciplinary actions against concerned doctors and related health care workers. To avoid the potential disciplinary actions, doctors tended to fill in a COD with the least ambiguity.

In Shanghai, the medical doctors were concerned about the potential medical treatment disputes when certifying death certificates. Violence against medical workers has been reported as an increasing problem in China [28]. Due to growing medical costs and mistrust of the judiciary system, some family members of patients who died from hospital-acquired infections blamed the attending doctors and claimed huge compensation payments from the hospital through disturbing regular clinical practice (known as *Yinao*) [29]. After *Yinao*, medical staff may be unfairly criticized or punished by their hospital. To avoid potential *Yinao*, physicians in Shanghai were less likely to certify pneumonia and septicemia (which may be related to nosocomial infection) as UCD in death certificates.

(d). Institutional influence on the governance and procedure of quality control of COD statistics

Differences in institutional structure on quality control may be another factor that affects the comparability of COD between the two cities. Clinical record review may reduce the likelihood of coding immediate conditions as UCD, as chronic diseases related to immediate conditions may be recorded in clinical records. When a clinical record review is conducted, physicians and coders obtain a diagnosis history of the decedent. Those immediate conditions are considered as the consequence of chronic diseases and coded as UCD. In Hong Kong, clinical record reviews are only conducted for persons who die from statutorily notifiable infectious diseases or those with insufficient information to determine UCD. However, these practices are conducted regularly in Shanghai, which may partly explain why the proportions of UCD by immediate conditions were lower in Shanghai than in Hong Kong.

Alternatively, renal failure and septicemia were regarded as mechanisms of death in the guidelines or training materials for physicians in Shanghai [21]. Moreover, in Shanghai, the training materials emphasized that pneumonia and bronchopneumonia may be accepted as complications of any disease [5]. Though a similar example was included in guidelines for certifying CODs in Hong Kong, no detailed information described the causal relationship between pneumonia and other diseases. More importantly, SCDC has the authority to review the quality of certifying CODs for physicians’ performance evaluations according to current regulations. In other words, the rules of the causal relationship between pneumonia and pre-existing chronic diseases are implemented more efficiently in Shanghai when compared with Hong Kong. Such differences may partly explain why doctors in Shanghai were more likely to certify pneumonia as a consequence of other diseases than those in Hong Kong.

## Discussion

Between these two Chinese cities with similar levels of longevity, our findings from the quantitative analysis show that UCD statistics of certain immediate conditions (e.g., septicemia, pneumonia, and renal failure) may not be comparable. The differentials in determining UCD were due to social and institutional factors in the process of death registration. First, the procedures of registering death certificates outside hospital affected dying persons’ preference of their location of death. The relatively lower proportion of deaths outside hospital in Hong Kong was attributed to the avoidance of complexity of the coronial process if they die outside hospital. Further, the possibility of lowering the property value of the deceased person’s residence further discouraged families from bringing their dying members home. Second, the difference in registering deaths outside hospital between the two cities contributes to the variation in the distribution of UCD. In Shanghai, reviewing clinical records as major evidence to determining COD for deaths outside hospital may increase the likelihood of pre-existing chronic diseases being considered as the UCD, but undervalue the role of immediate conditions. Third, the selection of UCD had considerable implications for the professional performance of doctors, which influenced doctors’ perceptions of the causal sequence of morbid events leading to death. Knowledge, attitudes, and perceptions of doctors in Hong Kong may have increased their likelihood of certifying septicemia, pneumonia, and renal failure as UCD. Doctors in Shanghai tended to hold the view that immediate conditions caused by pre-existing chronic diseases should not be coded as UCD. Their concerns about potential medical disputes further reduced the likelihood of certifying the immediate conditions as UCD. Fourth, institutional influence on the procedure of quality control of COD statistics affected the determination of UCD. Regular clinical record reviews in Shanghai may reduce the likelihood of coding immediate conditions as UCD.

Previous cross-regional research suggested that physicians’ and coders’ tendency to certify certain CODs varies across different areas [7, 10, 11]. These tendencies seem to be associated with physicians’ and coders’ perceptions of the causal sequence of morbid events leading to death [7, 30, 31]. For example, previous research suggested that physicians in Taiwan may be more likely to believe that the causal relationship between diabetes and immediate conditions is sufficiently strong, compared with counterparts in Sweden and Australia [7].

Building on existing knowledge, this study reveals how institutional factors (e.g., laws and regulations, health systems, and related implications of doctors’ performance under different medical training programmes and practice) influence physicians’ behavior related to death certification. Laws and regulations affect the processes of registering deaths outside hospital, thus affecting both preference of location of death and determination of UCD. Different regulations also affect the procedure for quality control of COD statistics, which diversifies determinations of UCD.

The dissimilarities in institutional structure between the two cities are partly inherited from their different political histories, affected and developed by different nations and their legal systems [21]. Hong Kong was colonized by the British in 1842, while Shanghai’s health system was historically influenced, first by Western countries during the International Settlement era and then by the Soviet Union after the establishment of the PRC. After the handover in 1997, the closer association between Hong Kong and other parts of Mainland China was unavoidable. Comparisons of health characteristics between Hong Kong and other Chinese cities became more popular, especially examining the causes of mortality risk difference, or evaluating the possible effects of health policy or health care services. Prior to attempting to interpret these inter-city differences in terms of etiological factors, it is necessary to examine the possible biases influencing the comparability of data [32].

The study has several policy implications for enhancing the comparability of COD data across different death registration systems. First, WHO needs to consider developing standard procedures for registering deaths outside hospital for countries that do not operate on a coronial system. Verbal autopsy has been widely used for ascertaining CODs without medical supervision [33]. Guidelines for certifying COD outside hospital may provide a standard that would combine information from clinical records with that from verbal autopsy questionnaires. In many developed countries with high prevalence of comorbidity, mobile computing devices, together with verbal autopsy questionnaires, can be used for registering deaths outside hospital [34]. In the late 2000s, Shanghai utilized a questionnaire for querying clinical records (if available) with a verbal autopsy questionnaire based on symptoms to investigate COD for cases where CODs were not clear, as a part of the Third National Retrospective Survey for Death Causes [35]. The further validation results suggested that the misclassification rate for UCD was lower than 5% [36]. It is suggested that such tools with mobile application are regularly used to certify CODs for deaths outside hospital in Shanghai. Concurrently, the methodologies of registering deaths outside hospital should be harmonized for better cross-regional comparability.

Second, to raise doctors’ awareness of the importance of the death certification practice and the value of death statistics, health authorities should provide standard guidelines and regular training for filling in the complete chain of COD. Standardized training could also increase doctors’ awareness of the importance of thoroughly completing a COD chain and the value of death statistics [11]. Also, completeness of a COD chain and accuracy of COD certification can be considered as a performance indicator for their clinical practice. In addition, more detailed certifying and coding standards should be included for deaths associated with immediate conditions (e.g., septicemia, pneumonia, and renal failure) in the Eleventh Revision of ICD. The current method of manually coding COD is ambiguous and allows doctors and coders to have different interpretations of the casual relationship between pneumonia and chronic conditions (e.g., wasting diseases, diseases causing paralysis) [5] (vol 2: p 40, see Appendix 1). Information on the length of disease onset, as well as syndromes and laboratory indicators of existing chronic diseases, should be taken into account when determining the causal sequence of morbid events leading to death.

Third, a unified automatic coding system should be established to improve the comparability of coding across different regions in China. Owing to the complexity of coding rules, heavy workload, and inter-rater disagreement among coders, it is difficult to monitor and assure the quality of COD statistics through manual coding. Therefore, a Chinese automatic encoding tool should be developed and implemented across different geographical regions in China.

Fourth, a globally accepted, standardized procedure for data review with validity checklists (e.g., clinical record review) should be developed. Clinical records are regarded as another relatively reliable source for validating COD when autopsy material is unavailable [37]. Given the increased availability of electronic clinical records, death certificates can be linked with clinical records. These linked data can facilitate the reviewing process of clinical records and assure data quality.

Fifth, given an aging population, a number of concurrent conditions often exist and contribute to a person’s death [38]. Analysis of UCD alone is inadequate to follow the prevalence of certain conditions over time using mortality statistics [11, 39]. Multiple causes of death (MCD) data should be included in the preparation of mortality statistics in areas experiencing population aging.

Several limitations of the present study should be noted. This study does not quantify the social and institutional effects of the differences in reporting certain cause-specific mortality. Different levels of reported cause-specific mortality could be partly associated with the epidemiological profiles of diseases. However, life expectancies in the two cities were both above 80 years in the study period. This suggests that the two cities were at the fourth stage of Epidemiologic Transition, “the Age of Delayed Degenerative Diseases,” and might have similar patterns of COD according to the revised theory of Epidemiologic Transition [40]. We compared cause-specific mortality in the two cities with selected East Asian and Western populations (Appendix 2) with similar levels of life expectancy and data quality [41]. The ratio of standard deviation to mean (SD/M) across nine populations for all-cause and malignant neoplasm mortality were close to zero (at 0.1). However, the corresponding figures for pneumonia, chronic lower respiratory disease, renal failure, and septicemia were comparatively higher. This suggested that those latter causes of death may be less comparable across different countries. Additionally, it was shown that ASDRs for pneumonia, renal failure, and septicemia in Hong Kong were the highest compared to others, while the corresponding figures in Shanghai were the lowest. The huge differentials in mortality risk by pneumonia, chronic lower respiratory disease, renal failure, and septicemia between Hong Kong and Shanghai and their social and institutional explanations may partly explain the data incomparability between the two cities.

Apart from those mentioned, this analysis only adjusted for age and sex as confounding variables, while other possible confounding factors (e.g., ethnicity, socioeconomic status, and family structure of deceased person) [2, 15] were not considered due to data unavailability. Second, due to the lack of information on location of death for individual records in Hong Kong, this factor was not included in the logistic regression analysis. Third, mortality from external causes in Hong Kong was underestimated for individual records due to the reporting delay from the coronial system. Fourth, due to the unavailability of data, we did not separate the analyses for number of deaths outside hospital that were certified by doctors in Hong Kong. Fifth, previous research suggests that the use of MCD can provide complementary information to demonstrate how certifying and coding affect the selection of UCD [39]. We did not conduct analyses on MCD as official MCD data are not available in Hong Kong. Sixth, differences in the coding rules of ICD-10 between the two cities may influence the validity of this analysis. In a recent official report [25], deaths by pneumonia, diabetes, and chronic kidney diseases through automated ICD-10 coding were found to have 1%–2% deviations when compared to those by manual coding. The different ICD-10 coding methods between Hong Kong (ACME) and Shanghai (manual) may affect the distribution of UCD, but this study did not assess its possible impact due to data unavailability.

In the future, it is recommended that a detailed validation study for COD statistics is conducted in Hong Kong. This future study could use external panel review of high-quality clinical records as a gold standard [42]. Such a validation study can help to further understand the implications of the various COD certification practices as evidenced from the qualitative research.

## Conclusion

In conclusion, variations in social and institutional factors were related to the processes of certifying and registering deaths in Hong Kong and Shanghai. These may contribute to the differences in the reporting of particular cause-specific mortality rates in these cities. Interpretation of regional differences in officially reported mortality statistics should take the social and institutional influence on death certification practices into account. To improve regional data comparability, health authorities should develop standard procedures with verbal autopsy questionnaires for registering deaths outside hospital; provide standard guidelines and training for filling in complete COD chains to raise certifiers’ and coders’ awareness of death certification practices; develop a unified automated coding system for COD, consolidate a standard procedure for data review and validity checks, and disseminate information on both UCD and MCD in mortality statistics.

## Appendix 1

Coding guidance related to pneumonia in “International Statistical Classification of Diseases and Related Health Problems Tenth Revision,” Volume 2 Instruction manual, 2010 Edition: 40.

Lobar pneumonia, unspecified (J18.1) should be considered an obvious consequence of dependence syndrome due to use of alcohol (F10.2). Any pneumonia in J12–J18 should be considered an obvious consequence of conditions that impair the immune system. Pneumonia in J15.0–J15.6, J15.8–J15.9, J16.8, J18.0 and J18.2–J18.9 should be considered an obvious consequence of wasting diseases (such as malignant neoplasm and malnutrition) and diseases causing paralysis (such as cerebral haemorrhage or thrombosis), as well as serious respiratory conditions, communicable diseases, and serious injuries. Pneumonia in J15.0–J15.6, J15.8–J15.9, J16.8, J18.0 and J18.2–J18.9, J69.0, and J69.8 should also be considered an obvious consequence of conditions that affect the process of swallowing. Pneumonia in J18.- (except labour pneumonia) reported with immobility or reduced mobility should be coded to J18.2.

## Appendix 2

**Table 5:** Age-standardized death rates (ASDR) in Hong Kong (2005–2008) and Shanghai (2005–2007), compared to selected East Asian and Western Populations (2008)

Cause	Australia	England & Wales	France	Germany	Japan	Korea	Sweden	Hong Kong	Shanghai	SD^a^	Mean^b^	Ratio (SD/M)
Septicemia (A40-A41)	3.0	1.9	3.0	4.0	2.8	2.5	3.5	6.3	0.02	1.7	3.0	0.6
Malignant neoplasms (C00-C97)	118.7	132.4	127.7	124.4	109.5	117.9	112.1	118.6	123.3	7.2	120.5	0.1
Diabetes mellitus (E10-E14)	10.5	4.6	7.5	10.5	4.3	17.8	8.7	4.7	15.1	4.8	9.3	0.5
Ischemic heart diseases (I20-I25)	56.8	63.8	25.2	63.6	21.8	22.4	65.9	36.0	40.2	18.7	44.0	0.4
Cerebrovascular diseases (I60-I69)	26.9	33.6	20.0	28.6	33.9	49.6	31.1	29.8	74.1	16.2	36.4	0.4
Pneumonia (J12-J18)	3.8	19.8	6.1	9.8	26.4	10.2	7.7	37.6	2.1	11.9	13.7	0.9
Chronic lower respiratory diseases (J40-J47)	15.8	22.4	6.0	12.9	4.7	13.4	12.0	17.5	38.8	10.1	15.9	0.6
Renal failure (N17-N19)	5.8	2.1	3.6	6.0	5.4	6.4	2.4	11.2	1.2	3.0	4.9	0.6
External causes (V01-Y89)	32.9	24.3	37.9	24.6	35.7	54.1	32.9	21.6	28.6	9.8	32.5	0.3
All causes	380.5	442.7	395.6	441.6	339.3	440.1	402.2	357.7	408.1	37.2	400.9	0.1
Life expectancy at birth (years)	81	80	81	80	83	80	81	82	81	--	--	--

^a^: standard deviation for ASDR in nine listed populations.

^b^: mean for ASDR in nine listed populations.

Data Sources:

ASDR in Australia, England & Wales, France, Germany, Japan, Korea, and Sweden was from WHO mortality database. Life expectancy was from the World Bank Data, http://data.worldbank.org/indicator/SP.DYN.LE00.IN?name_desc=true. Accessed 30 Oct 2016.

## Abbreviations

ACME: Automated Classification of Medical Entries
COD: Cause of death
GBD: Global Burden of Disease
ICD: International Classification of Diseases
SCDC: Shanghai Center for Disease Control and Prevention
UCD: Underlying cause of death
WHO: World Health Organization
